# Environmental and Genetic Factors in Autism Spectrum Disorders: Special Emphasis on Data from Arabian Studies

**DOI:** 10.3390/ijerph16040658

**Published:** 2019-02-23

**Authors:** Noor B. Almandil, Deem N. Alkuroud, Sayed AbdulAzeez, Abdulla AlSulaiman, Abdelhamid Elaissari, J. Francis Borgio

**Affiliations:** 1 Department of Clinical Pharmacy Research, Institute for Research and Medical Consultation (IRMC), Imam Abdulrahman Bin Faisal University, Dammam 31441, Saudi Arabia; nbalmandil@iau.edu.sa; 2 Department of Genetic Research, Institute for Research and Medical Consultation (IRMC), Imam Abdulrahman Bin Faisal University, Dammam 31441, Saudi Arabia; dalkroud@gmail.com (D.N.A.); asayed@iau.edu.sa (S.A.); 3 Department of Neurology, College of Medicine, Imam Abdulrahman Bin Faisal University, Dammam 31441, Saudi Arabia; Dr.aaalsulaiman@gmail.com or aasulaiman@iau.edu.sa; 4 Univ Lyon, University Claude Bernard Lyon-1, CNRS, LAGEP-UMR 5007, F-69622 Lyon, France; abdelhamid.elaissari@univ-lyon1.fr

**Keywords:** neurodevelopmental disorders, environment, genetics, autism, synaptic transmission

## Abstract

One of the most common neurodevelopmental disorders worldwide is autism spectrum disorder (ASD), which is characterized by language delay, impaired communication interactions, and repetitive patterns of behavior caused by environmental and genetic factors. This review aims to provide a comprehensive survey of recently published literature on ASD and especially novel insights into excitatory synaptic transmission. Even though numerous genes have been discovered that play roles in ASD, a good understanding of the pathophysiologic process of ASD is still lacking. The protein–protein interactions between the products of *NLGN*, *SHANK*, and *NRXN* synaptic genes indicate that the dysfunction in synaptic plasticity could be one reason for the development of ASD. Designing more accurate diagnostic tests for the early diagnosis of ASD would improve treatment strategies and could enhance the appropriate monitoring of prognosis. This comprehensive review describes the psychotropic and antiepileptic drugs that are currently available as effective pharmacological treatments and provides in-depth knowledge on the concepts related to clinical, diagnostic, therapeutic, and genetic perspectives of ASD. An increase in the prevalence of ASD in Gulf Cooperation Council countries is also addressed in the review. Further, the review emphasizes the need for international networking and multidimensional studies to design novel and effective treatment strategies.

## 1. Introduction

Autism spectrum disorder (ASD) is a common heterogeneous neurodevelopmental condition that has three main characteristics: Language delay, social interaction and communication impairment, and repetitive actions or interests [1]. Furthermore, in addition to these main symptoms, autistic people often have comorbidities such as intellectual disability, gastrointestinal disorders, epilepsy, immune disorders, and sleeplessness. The word “autism” was first used by Kanner in 1943. Over the years, the diagnosis has been extended to include multiple types of autism. Three subtypes were described, based on the severity of clinical conditions: Autism spectrum disorder encompasses autism, Asperger’s disorder, autistic disorder, childhood disintegrative disorder, childhood autism, and pervasive developmental disorder not otherwise specified [2]. The autistic child is typically diagnosed before 3 years of age [1]. In the manual of psychiatric diagnosis, autism was not considered a distinct disorder, nor was it considered by most to be biologically based until the 1980s [3]. This review provides a comprehensive literature survey on ASD, with a particular focus on data obtained by investigators in the Gulf region. This might ultimately help to improve the assessment and treatment of ASD on the Arabian Peninsula.

## 2. Diagnosis

Diagnosis of ASD is currently established by means of criteria set forth in the Diagnostic and Statistical Manual of Mental Disorders, version 5 (DSM-5) [4]. The DSM-5 changed the diagnostic criteria by eliminating ASD subtypes and generating a new category known as ASD [5].

Physicians have a crucial role in the early diagnosis of ASD [6]. The onset of the disorder occurs typically by the age of 3, although symptoms may not manifest until school age or later [7]. Even though autism is a well-known neurobehavioral and developmental condition, it lacks satisfactory screening tools, leading to delays in diagnosis and therapeutic advancements. Current screening tools include the infant toddler checklist (ITC), which is used in the screening of children ages 9 months to 2 years, and the modified checklist for autism in toddlers—revised (M-CHAT-R), intended for children between 16 and 30 months [8]. Intellectual disability, gastrointestinal disorders, attention and immune deficits, epilepsy, sensory sensitivities, depression, and anxiety are known to be associated with ASD [5].

## 3. Public Health Impact

ASD is considered as one of the most serious conditions in the U.S., with caregiver, family, and financial burdens. ASD costs are estimated to be approximately $250 billion annually in the U.S. Moreover, it is suggested that by 2025, ASD costs will rise to over $450 billion. With this huge financial need and increased prevalence, ASD has become an economic burden on both families and society [5]. In Saudi Arabia, a study that aimed to explore the characteristics of ASD and its impact on the families of autistic children reported that 59.9% of families claimed that their autistic child negatively affected their lifestyle socially and economically (60.4%), their family relationships (53.5%), and the siblings’ quality of life (62.6%), all of which leads to parental distress (88.5%) [9].

## 4. Epidemiology

The prevalence of ASD ranges from about 25 to 110/10,000 children [10,11]. The incidence rate of ASD in family members of a child with autism is 2–8% higher than in the general population. Moreover, it is reported that ASD occurs more frequently in males than females [12]. The prevalence of ASD has been increasing worldwide due to the broadening of diagnostic criteria and wider public awareness of the disorder. The prevalence of autism is variable; the US reported a median of 21.6 per 10,000, Europe reported a median of 18.75 per 10,000, and China reported a lower median of 11.6 per 10,000 [13]. Reports suggest an increase in the prevalence of ASD in Gulf Cooperation Council (GCC) countries, and it is considered as one of the most common disabilities. However, studies of the condition in the GCC countries and Asia in general are rare. Prevalence studies were conducted in the UAE, Saudi Arabia, Oman, and Bahrain (Table 1). The prevalence of ASD was 1.4 per 10,000 in Oman, 29 per 10,000 for PDD (pervasive developmental disorder) in the UAE, and 4.3 per 10,000 in Bahrain [13]. In Saudi Arabia, 42,500 autism cases were diagnosed, and many more cases remained undiagnosed in 2002 [14]. Moreover, a study done in 2007 revealed that the prevalence of ASD was 1/167 in Saudi Arabia [15]. Most recently, a study conducted in 2013 in Taif (Saudi Arabia) reported that the prevalence of ASD in males (0.031%) was greater than in females (0.004%) [16]. The prevalence of ASD among Saudi Arabians has not been determined.

## 5. Etiology

The causes of autism are still poorly understood. Etiological theories have changed throughout the years. Until the 1970s, faulty child-rearing was thought to be the cause, but this theory has been rejected. Currently, ASD is considered to be a multifactorial disorder caused by genetic, epigenetic, and environmental factors [1].

## 6. Genetic Factors

Family and twin studies have demonstrated that approximately 10% of children are diagnosed with ASD as a part of other genetic or neurological disorders, such as fragile X syndrome, tuberous sclerosis, phenylketonuria, or congenital infections secondary to rubella virus and cytomegalovirus [21]. Moreover, if the family already has an autistic child, the possibility of having another child with autism increases 25 times in comparison to the general population [22].

Twin studies have suggested that monozygotic (identical) twins have 60–90% concordance rate of having autism, while dizygotic (nonidentical) twins have a 0–24% decreased risk [23]. Furthermore, the risk of ASD may be increased by structural variations or mutations [22].

### 6.1. Genetic Studies of ASD

Since the disorder is heterogeneous, it is challenging to precisely identify the underlying genetics. Numerous networking approaches are required to investigate the loci that are responsible for ASD. The hypothetical risk factors for neurodevelopmental disorders result from health behaviors linked with socioeconomic factors, such as the use of recreational drugs or abuse of alcohol, parental mental health disorders, and genetic influences [24]. Few studies offer a genetic understanding of ASD, and each has its advantages and limitations. The most common approaches are cytogenetic analysis association and linkage analysis studies, copy number variation (CNV), and, most importantly, DNA microarray analysis, as well as whole-exome sequencing analysis and transcriptomic analysis [2].

### 6.2. Cytogenetic Studies

One of the classical genetic approaches is cytogenetic analysis, which aims to detect the contribution of chromosomal aberrations in children’s disorders. The hypothesis is that autism is an outcome of rare de novo or sporadic mutations and that it is not usually inherited [2,25]. Various candidate genes and chromosomal aberrations associated with ASD have been identified using cytogenetic studies [26,27,28,29,30].

### 6.3. Copy Number Variation (CNV) Analysis

CNV is a modern cytogenetic technique, particularly used to identify indels of DNA fragments larger than 50 kb [2,31]. Numerous findings have highlighted the importance of CNV and autism [31,32,33,34,35,36]. CNVs around *SHANK2, DLGAP2, SYNGAP1, UBE3A, DPP10, PLCB1, TRPM1, NRXN1, FHIT, HYDIN, PARK2, RFWD2, AUTS1, AUTS5, IMMP2L–DOCK4, FBXO40*, and *DDX53–PTCHD1* loci or genes were found to be associated with disrupting functional genes, influencing the ubiquitin pathways and functional convergence in autistic children [31,32,33,34,35,36,37]. *NLGN1, NLGN2, NLGN3, NLGN4*, and *NLGN4Y* genes play roles in synapse formation and synaptic function [38,39].

### 6.4. Linkage and Association Studies

Different from cytogenetics, linkage analysis aims to detect the transmission of autism genetic loci in families of affected individuals. Association studies based on case-control and family studies have examined the changes between groups in terms of alleles and genotype frequencies. Microsatellite markers or single nucleotide polymorphisms (SNPs) were chosen based on linkage studies. Since association studies are used to detect susceptible alleles in an increased number of patients compared to controls, it fails to detect rare mutations [40,41,42,43,44].

### 6.5. Microarray Analysis

Rapid improvements and advancements in microarray technology have led to an improved ability to detect submicroscopic chromosomal abnormalities. This technology allows identification of uncommon and de novo mutations. In addition, whole-exome arrays and newly developed microarrays have been used to identify de novo mutations in multiple disorders. Since protein coding genes (exons) are responsible for 85% of mutations in disorder-related traits, whole-exome sequencing plays a role in identifying rare mutations in heterogeneous disorders when linkage studies are impossible. Furthermore, due to the complexity of the disorder, studies that have used data of gene expression to evaluate mRNA of specific genes have led to better understanding of ASD. In recent years, many studies have tried to identify predictive biomarkers for ASD, but no such biomarker has thus far proven clinically useful [2,40,41,42,43,44,45,46,47,48]. A study conducted in 2017 used whole-exome sequencing on 19 trio subjects from Saudi families. The study revealed 47 unique rare variants in 17 trios, including 38 that were newly discovered. The variants were in 15 ASD candidate genes, including 5 genes that have not been included in any human conditions (*GLT8D1*, *OR6C65*, *HTATSF1*, *DDX26B*, and *ITIH6*), and the rest of the variants were linked with ASD or other neurological conditions, such as schizophrenia and intellectual disabilities. Most of the variants were either X-linked or autosomal recessive [49].

### 6.6. Genome-Wide Association Studies

Genome-wide association studies (GWAS) in ASD and studies of copy number variations (CNVs) have resulted in a large list of potentially important genes; however, a satisfactory theory about the underlying pathophysiological process of ASD has yet to be formulated [50,51,52,53,54]. Below is the list of GWAS conducted over the past few years. The leading successful study was performed by Wang et al. [52] and Glessner et al. [33], involving around 3000 individuals of European origin from more than 700 families and 6491 controls and 1204 cases. Using 6 SNPs between *CDH9* and *CDH10*, the study reported significant association signals with the best significant SNP, rs4307059 (*p* = 3.4 × 10^−8^), located in chromosome 5p14.1. The associated signals were reproduced with *p*-values from 7.4 × 10^−8^ to 2.1 × 10^−10^ in two independent cohorts. Later, Kerin et al. [53] and Wang et al. [52] continued the work and described 3.9 Kb noncoding RNA, *MSNP1* antisense transcribed from the chromosome 5p14.1 region using a tiling array. Glessner et al. [33] conducted a study on a dataset of approximately 430 Caucasian families with autism using Illumina Human 1M beadchip. A total of 96 SNPs were found to be associated with autism risk (*p* < 0.0001), and these were validated with another dataset with 487 families of European origin with autism and genotyped on 550K Illumina beadchip. The same region that was mentioned by Wang et al. [52] (5p14.1) showed significant association in both datasets. Combined dataset analysis of all SNPs in this region showed a total of 8 SNPs with enhanced *p*-values (3.24 × 10^−4^ to 3.4 × 10^−6^). An association and linkage mapping study was performed by Weiss et al. [55] and Glessner et al. [33] using 500,000 SNPs in a group of multiplex autism families (1553 affected offspring). The study reported significant linkage on chromosome 20p13 and one significant (*p* = 2 × 10^−7^) SNP identified on chromosome locus 5p15 between *TAS2R1* and *SEMA5A* with autism. Anney et al. [56] and Glessner et al. [33] confirmed that the association signal for variant rs4141463 (*p* < 5 × 10^−8^) was positioned in *MACROD2*. The small sample size slightly affected the association with autism less than the threshold (*p* < 5 × 10^−8^). However, the study yielded significant signals within five genes, *KIAA0564, POU6F2, PLD5, TAF1C*, and *ST8SIA2*. Cho et al. [57] and Glessner et al. [33] did a GW scan using Affymetrix SNP array 5.0 to analyze 42 Korean ASD patients and detected candidate SNPs in chromosome 11, rs11212733 (*p* = 9.76 × 10^−6^) and rs7125479 (*p* = 1.48 × 10^−4^), as biomarkers of language delay among autism subjects through multifactor dimensionality reduction and transmission disequilibrium test. Glessner et al. [33] and Anney et al. [56] reported a study showing a million SNPs covering up the genome, but not any candidate risk SNP with ASD. The SNP rs1718101 showed the lowest *p*-value in the *CNTNAP2* gene, previously reported as susceptible to the development of autism [58]. An association study between genomic loci and individual assessment items was conducted by examining up to 2165 participants with identified *NELL1* (*p* = 2.91 × 10^−7^), *NOS2A* (*p* = 8.12 × 10^−7^), and *KCND2* (*p* = 3.05 × 10^−8^) genes with autism risk. In Saudi Arabia, the Saudi Human Genome Program (SHGP), supported by King Abdulaziz City for Science and Technology (KACST), was established in 2013, and an association study on autism by Al-Mubarak et al. [49] revealed various genes with autism risk.

## 7. Synaptic Genes

**Figure 1:**
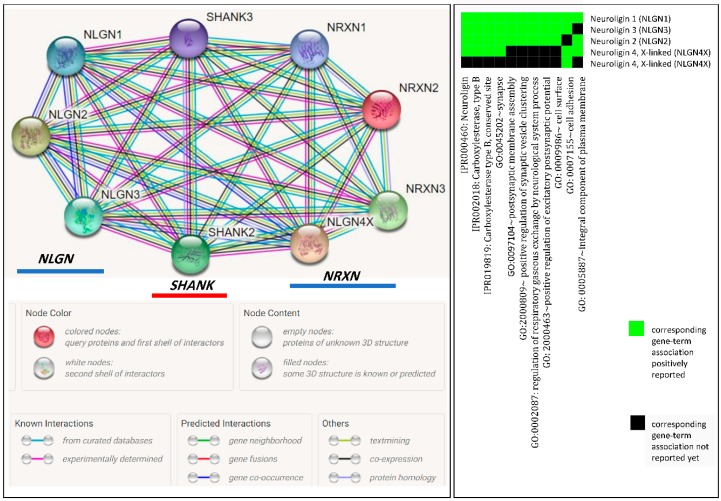
(**Left**) STRING results of protein–protein interactions between *NLGN*, *SHANK*, and *NRXN* synaptic genes. (**Right**) Functional annotation clustering from DAVID.

Mutations in synaptic genes, such as postsynaptic cell adhesion molecules neuroligins (*NLGN4X* and *NLGN3*) and postsynaptic scaffolding proteins (*SHANK2* and *SHANK3*) and presynaptic cell adhesion molecule neurexin 1 (*NRXN1*), are considered to be the most reported genetic abnormalities associated with ASD. These mutations are nonspecific to ASD and are also associated with other neuropsychiatric disorders, including schizophrenia and Alzheimer’s disease. Since ASD shares conditions with these neuropsychiatric diseases, mutations in synaptic genes are still considered to be common contributors to neuropsychiatric illnesses [59,60,61]. Neurexins (*NRXN*s), which are cell adhesion molecules located in the presynaptic cells, are ligands of neuroligins and thus facilitate a network of interrelated molecules from the trans-synaptic interaction between NRXNs and NLGNs and the postsynaptic density complex that includes SHANK3. Neuroligins (NLGNs) are cell adhesion molecules in postsynaptic cells have been shown to bind to postsynaptic scaffolding protein (SHANK3) and are mandatory in the glutamatergic synapse, as they are necessary for β-NRXN binding and synaptic activity, and mutations in NLGNs impair synaptic function. Results from STRING (https://string-db.org/) showed the presence of protein–protein interactions between *NLGN*, *SHANK*, and *NRXN* synaptic genes, and functional annotation clustering from DAVID (https://david.ncifcrf.gov/home.jsp) indicated the possibility of ASD due to dysfunction in synaptic plasticity (Figure 1).

### Mechanism of Action

Although neurotransmitter and neurochemical abnormalities have been implicated in ASD with or without comorbidities, several literature reviews suggest neurochemical perturbations particularly affect serotonin, gamma aminobutyric acid, dopamine, and epinephrine [38,62,63,64,65], of which GABAergic, glutaminergic, and dopaminergic factors might play a role in ASD and sleep disturbances in affected children. Minimizing or reversing these perturbations using antiepileptic drugs tailored to these patients may prove to be a psychotropic success [62,63,64,65].

**Figure 2:**
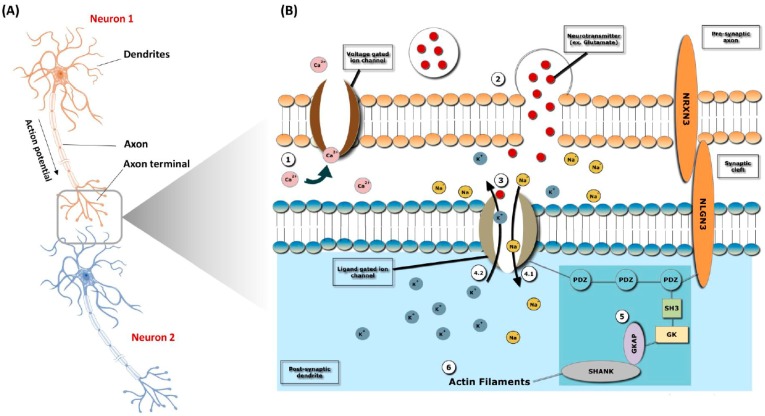
Synaptic transmission pathway (STP) of ASD. (**A**) Initiation of STP in a neuron upon receiving signals from another neuron. (**B**) Action potential and conversion of signals into chemical message: (1) Binding of cell adhesion molecules, *NRXN1* (presynaptic axon) and *NLGN3* (postsynaptic dendrite). Calcium (Ca^2+^) enters into the cell. (2) Formation and release of vesicles containing neurotransmitters into the synaptic cleft. (3) Opening of the ligand gated ion channels and entry of sodium (Na^2+^) into the cell. (4) Leaving potassium (K^+^) out of the cell. (4) Activation of SHANK protein. (6) Molecular organization of postsynaptic density and modulation of postsynaptic signal transduction.

Neurotransmission, also known as synaptic transmission, is a process by which neurons communicate by releasing signaling molecules known as neurotransmitters. The process initiates when a neuron receives signals from another neuron through dendrites. When there is enough signal and it reaches the −55 mV threshold, an electrical signal called the potential action moves through the axon until it reaches the axon terminal (Figure 2A). As the action potential reaches the axon terminal, the signal is converted into a chemical message. Cell adhesion molecules *NRXN1* (presynaptic axon) and *NLGN3* (postsynaptic dendrite) bind to each other. The voltage-gated calcium (Ca^2+^) channel opens, and calcium enters the cell (Figure 2B). Vesicles containing neurotransmitters are then formed and fuse with the membrane, releasing neurotransmitters into the synaptic cleft (Figure 2B). Neurotransmitters bind to ligand-gated ion channels and cause them to open, allowing sodium (Na^2+^) to enter the cell and potassium (K^+^) to leave, depolarizing the region (Figure 2B). This activates a series of molecules leading to the activation of the SHANK protein, which is a scaffolding protein necessary for connecting neurotransmitter receptors and ion channels to actin cytoskeleton (Figure 2B). Actin filaments regulate the molecular organization of the postsynaptic density and modulate postsynaptic signal transduction (Figure 2B). If depolarization crosses the −55 mV threshold, an action potential is produced in the next neuron (Neuron2). Depolarizing actin filaments facilitate the docking and fusion of secretory granules at the plasma membrane [62,63,64,65].

## 8. Environmental Factors

Earlier, twin studies suggested that 80–90% of ASD is caused by heritable factors, with little environmental contribution. However, recent studies reported that 40–50% of variance is found by environmental factors. Moreover, recent twin studies suggest that both genetic and environmental factors contribute to the development of ASD [5]. These environmental factors are discussed in detail below.

### 8.1. Parental Age

A meta-analysis study performed by Wu et al. including 27 studies on the association between increased maternal and paternal age revealed that a 10-year increase in either maternal or paternal age increases the risk of having offspring with ASD by 18 and 21%, respectively [5].

### 8.2. Medication Use During Pregnancy

The evidence of a relationship between parental use of valproate (used for epilepsy and bipolar disorder) and autism was reviewed by Gentile [66]. Studies showed a robust indication for a relationship between maternal use of valproate and ASD. It is controversial to suggest that the use of antidepressants during pregnancy and autism are associated. Case-control studies and three cohort studies examined the use of selective serotonin reuptake inhibitors (SSRIs) among pregnant women and the probability of autistic offspring [67]. Results showed a 50% increase in the risk of autism in children of mothers who received SSRIs in pregnancy. However, an investigation comparing mothers with psychiatric conditions not exposed to SSRIs to those exposed to SSRIs revealed no significant increase in the risk of ASD in their offspring. The author suggested that the relationship between autism and SSRIs is attributable to confounding factors by indication.

### 8.3. Maternal Smoking and Alcohol Consumption

Even though smoking and alcohol consumption during pregnancy lead to adverse neonatal outcomes, evidence from the existing literature suggests that prenatal use of these substances is not related to ASD risk. A meta-analysis by Rosen et al. listed 15 studies that showed no significant association between maternal smoking during pregnancy and the chance of autism in children [68].

### 8.4. Vaccination 

Vaccination is a very hot topic that has been discussed globally on many different occasions. There are various studies and many research papers concerning its relationship to autism; some deny the hypothesis that it plays a role in the development of ASD and others support that relationship. In 1998, there was public concern that the measles, mumps, and rubella (MMR) vaccination was related to autism. This led to a decrease in MMR vaccination of children [69], resulting in a measles outbreak, which led to an increase in the amount of research worldwide reconfirming the relationship. Madsen et al. [70] examined Dutch children from January 1991 until December 1998 and revealed no relationship between time since vaccination, age at time of vaccination, and development of ASD. In addition, a meta-analysis performed by Taylor et al. [71] examined the association between childhood vaccination and ASD. The study reported that there was no proof of a greater risk of autism in vaccinated children.

On the contrary, since vaccines contain aluminum nanoparticles used as an adjuvant, it is controversial to suggest that vaccination has an effect on the development of autism. Wei et al. [72] described depressive symptoms, not symptoms of ASD. Further, aluminum microparticles might trigger (brain) inflammation, and this is known to promote depressive symptoms. It is therefore doubtful that preclinical experiments that apparently support the contention that vaccination might cause ASD are correctly interpreted. Moreover, in contrast to the situation in depression, the evidence that interleukin-6 (IL6) levels are increased in patients with ASD is extremely sparse. The French scientists Gherardi et al. [73] performed a study and found that aluminum contained in vaccines did travel to the brain, leaving us with the ultimate question of whether vaccination is or is not related to autism.

## 9. Management Strategies

When a physician sees a patient with the possible diagnosis of ASD, there are few objective laboratory tests, hematologic or otherwise, to aid in the diagnosis. This poses a challenge to practicing physicians. The role of electroencephalography (EEG) and EEG monitoring may prove helpful in unveiling unforeseen diagnoses or comorbidities, which aids in the selection of further beneficial and proven therapies, including antiepileptic drugs [62]. Neuroimaging, particularly magnetic resonance imaging (MRI) studies, whether structural or functional, may also be useful in either unraveling typical changes of a specific comorbidity or confirming volume loss in the deep gray matter, particularly in the caudate and striatum, which is frequently seen in ASD patients [62,74,75]. ASD is a long-lasting disorder. Although there is no current cure, several medications have been used to manage associated behavioral problems. Researchers have shown that the most effective treatment is the use of early behavioral therapy, which improves the functioning of affected children [76]. It mainly focuses on improving language, social interaction, and imitation skills, and adequate behavior. These treatments include treatment and education of autistic and related communication handicapped children (TEACCH) and applied behavioral analysis (ABA). Since there is still no cure for ASD, families are trying to figure out alternative therapies, such as the use of large doses of vitamins and dietary supplements, chelation therapy (detoxification), and hyperbaric oxygen therapy, to reduce the severity of symptoms [62].

### Medications

Medications may be prescribed to manage symptoms associated with ASD, but they may exhibit side effects and need to be prescribed only by a doctor or a person who specializes in the condition. These medications are customarily given by a neurologist or psychiatrist (Table 2) [77]. Several Food and Drug Administration (FDA)-approved medications are used to treat ASD-related traits such as instance irritability, aggression, hyperactivity, etc. (Table 3) [78].

Cobalamin’s purpose in the body is to enhance the function of the brain and nervous system. There are multiple types of cobalamin, but the most critical type is methyl B12, since it stimulates biological pathways that provide energy to the brain. Autistic children are reported to have decreased brain levels of methyl B12 [79]. Therefore, Hendren et al. [80] performed a study to determine if methyl B12 injection could improve symptoms of autism. The study compared two autistic groups, one injected with methyl B12 and the other injected with saline. Results showed that children with ASD who were treated with methyl B12 had significant improvement in symptoms compared to children injected with placebo or saline. Although the study had limitations, such as sample size, the results were notable, assuming a new discovery that brain levels of methyl B12 are reduced in autism [79].

## 10. Impact on Families

A diagnosis of autism not only affects the life of the autistic child but also affects the lives of family members in different ways. The family of an autistic child will encounter difficulties, and everyday activities will change, since the child needs extra attention from his or her parents. A study done by Banach et al. [91] found that after their child was diagnosed as autistic, 43% of parents felt grief and loss, 29% felt shock, 52% felt relieved, and 10% felt self-blame. Upon the initial diagnosis of their child’s behavior, adaptation to a new lifestyle, and complexity in finding access to services, parents mostly suffered from stress [91]. Such stress may affect the parents’ marital relationship, increase their financial burden, and lead to their isolation from others. Studies have pointed out that mothers suffer from stress more than fathers in relation to autistic children, since they are the children’s primary caregivers [92]. The main stressful issue for parents is that the autistic child is unable to express his/her basic needs. This disappoints both the parents and the child and results in an aggressive attitude by the autistic child, as the parents will not be able to know if their child is sick, hungry, tired, sad, or mad, especially if the child is nonverbal. Parents may hesitate to take their child to relatives or friends’ houses or any public places, since some people in the community may not understand the child’s behavior and be sensitive. This gives parents a feeling of isolation from family, friends, and the community (Autism Society, 2011). The financial aspect is considered to be an important issue. Autistic children need special services to assist in their care, which causes financial stress for the parents. Moreover, if one of the parents decides to quit his/her job to help take care of the autistic child, this will obviously lead to financial stress, since the other parent will be responsible for supporting the entire family [93]. In addition to parents, siblings are also impacted when an autistic child is diagnosed. Siblings may feel embarrassed around peers. They may also feel jealous, since their parents may need to spend more time with the autistic brother/sister. They may also feel frustrated over not being able to understand their autistic brother/sister.

In the UK (2015), a sample of 1047 parents completed a survey assessing their experiences and views concerning the process of attaining a diagnosis of ASD. Results demonstrated that most families frequently waited for 1 year before reaching out for professional help. This may be due the fact that most of these families were not satisfied with the diagnosis process. The level of family satisfaction was determined by several factors, such as time needed for diagnosis and information provided at diagnosis [94].

A study on the impact of autistic children on their families was done in Saudi Arabia based on a self-administered questionnaire of 227 families. Most parents (88.5%) stated that autism centers contributed to the treatment of autistic children, but only 31.7% had their autistic children regularly attending autism centers. Speech therapy is the most common therapy used, followed by behavioral and pharmacological therapies. Some families pointed out the use of traditional therapy, such as cauterization and/or roqia (a form of burning a part of the body). Others preferred dietary therapy, such as a gluten-free, casein-free, and/or sugar-free diet [9,95].

## 11. Conclusions

ASD is characterized by three features: Language delay, impaired social and communication interactions, and repetitive behavior or interests. It is one of the most common disorders worldwide and is caused by environmental and genetic factors. The diagnosis is based on the DSM-5 criteria. No objective diagnostic tests are available. EEG and neuroimaging play a major role in the diagnosis and in the selection and follow-up of therapeutic responses. Psychotropic and antiepileptic drugs are currently available as effective pharmacological treatments. Even though numerous genetic studies have identified various ASD-associated genes and functional convergence, researchers still cannot determine the ASD-causing genes and their impacts on the development of ASD. Further international networking and multidimensional studies are needed to reveal the actual facts in order to design effective treatment strategies.

## Figures and Tables

**Table 1:** Prevalence of autism spectrum disorder (ASD) in Gulf Cooperation Council countries.

Country	Prevalence	Reference
Oman	1.4 per 10,000	[17]
UAE	29 per 10,000	[18]
Bahrain	4.3 per 10,000	[19]
Saudi Arabia	No proper prevalence to date, but several studies mentioned the number of autistic patients	[14,20]
Kuwait	None	None
Qatar	None	None

**Table 2:** Role of electroencephalography (EEG) and antiepileptic drugs (AEDs) in ASD.

EEG Findings *	AED Used	Behavioral Improvement	Reference
Normal	Valproic acid	Present	[81]
Abnormal	Valproic acid	Present	[82]
Abnormal	Valproic acid	Present	[83]
Abnormal	Valproic acid	Present	[84]
Abnormal	Valproic acid	Present	[85]
Normal	Valproic acid	Present	[86]
Abnormal	Carbamazepine	Not present	[87]
Abnormal	Carbamazepine	Present	[88]
Abnormal	Carbamazepine	Present	[89]
Abnormal	Lamotrigine	Present	[90]

* Abnormal: presence of focal spikes and sharp waves [77].

**Table 3:** Autism spectrum disorder medications (modified from LeClerc et al. [78]).

Condition	Drugs
Irritability and aggression	Risperidone, aripiprazole, clozapine, haloperidol, sertraline
Aberrant social behavior	Oxytocin, secretin
Hyperactivity and inattention	Methylphenidate, venlafaxine
Repetitive behaviors	Fluoxetine, citalopram, bumetanide
Cognitive disorders	Memantine, rivastigmine
Insomnia	Mirtazapine, melatonin
